# A GRU-based traffic situation prediction method in multi-domain software defined network

**DOI:** 10.7717/peerj-cs.1011

**Published:** 2022-06-23

**Authors:** Wenwen Sun, Shaopeng Guan

**Affiliations:** 1 School of Information and Electronic Engineering, Shandong Technology and Business University, Yantai, Shandong, China; 2 School of Electronic and Information Engineering, Huaibei Institute of Technology, Huaibei, Anhui, China

**Keywords:** GRU, Traffic situation prediction, Multiple domains, Salp swarm algorithm, Software-defined networking

## Abstract

With the continuous development and improvement of Software-Defined Networking (SDN), large-scale networks are divided into multiple domains. Each domain, which is managed by a controller, forms multi-domain SDN architecture. In multi-domain SDN, the dynamics and complexity are more significant, bringing great challenges to network management. Comprehensively and accurately predicting traffic situation in multi-domain SDN can better maintain network stability. In this article, we propose a traffic situation prediction method based on the gated recurrent unit (GRU) network in multi-domain SDN. We first analyzed the relevant factors that affect data traffic and control traffic and transformed them into a time series of actual situation values. Then, to enhance the prediction performance of GRU, we used the salp swarm algorithm to optimize the hyperparameters of GRU automatically. Finally, we adopted hyperparameter optimized GRU to achieve traffic situation prediction in multi-domain SDN. The experimental results indicate that the proposed method outperforms other traditional machine learning algorithms in terms of prediction accuracy.

## Introduction

Software-Defined Networking (SDN) is a new network architecture, which decouples the data plane and the control plane, and controls the network in a software-defined way. Therefore, it is more flexible and intelligent than a traditional network (Kreutz et al., 2014). The controller is the core component of the control plane and controls the entire network. The switches in the data plane are responsible for data forwarding (Xu, Wang & Xu, 2020). At the beginning of SDN design, a single controller was used to manage the entire network. Due to the small network scale at that time, a single controller could effectively manage the entire network (Hu et al., 2018). With the network scale expansion and business demand growth, the inherent defects of a single controller have become increasingly prominent, such as limited processing capacity of the controller and single point of failure (Wang et al., 2017). Therefore, large-scale networks need to be reasonably divided into multiple domains. Each domain, which is managed by a controller, forms a multi-domain SDN network architecture.

The multi-domain architecture improves the scalability and flexibility of the network. However, as a complex system, the state of the network is always dynamic and is affected by many factors, such as network attacks, disasters and emergencies. In addition, the network topology is complex, and frequent information exchanges and emerging new applications cause a surge in network traffic. In the multi-domain architecture, the dynamics and complexity are more significant. Therefore, the management of multi-domain SDN network faces huge challenges.

Network traffic situation prediction, based on the network traffic data within a period of time (*i.e.*, the situation values obtained by situation assessment), uses expert knowledge and theoretical methods such as data mining to predict the future network status. It can enable network managers to grasp the next development trend, provide an important basis for network resource allocation in time, and avoid network congestion to a certain extent (Roman, Lopez & Gritzalis, 2008; Arendt et al., 2016). Therefore, traffic situation prediction has become an important part of network management (Xie et al., 2018).

In traditional networks, the existing traffic situation prediction methods can be divided into two categories: linear methods and nonlinear methods. Among them, the linear methods mainly include auto regression (AR) (Lu et al., 2016), moving average (MA) (Feng & Shu, 2005), autoregressive moving average (ARMA) (Xu & Zeng, 2014), *etc*. As the network architecture becomes more complex, the characteristics of the time series data in the network exceed the comprehensible range of the Poisson distribution and Markov distribution model. Furthermore, the linear model is only suitable for time series prediction with stable characteristics. For time-series with non-stationary and non-linear characteristics, the linear model cannot fully reflect the complexity change, which makes the prediction accuracy low. The non-linear methods mainly include support vector machine (SVM) (Feng & Liu, 2011), radial basis function neural network (RBFNN) (Jiang et al., 2017; Wei, 2017), general regression neural network (GRNN) (Zhuo, Zhang & Gong, 2012), Elman network (Guoli, 2019) and Back Propagation Neural Network (BPNN) (Haviluddin & Alfred, 2014), *etc*. Compared with the linear methods, the nonlinear methods improve prediction accuracy. However, there are some limitations. Although SVM requires a small number of samples, its prediction accuracy is greatly affected by the key parameters which are difficult to determine. RBFNN can approximate arbitrary nonlinear functions, but it has disadvantages such as being easily trapped in local optima and difficult to determine the network structure. Most of the neurons in GRNN cannot be activated, which has higher requirements for training samples. Elman network and BPNN have disadvantages such as over-fitting and poor stability. In addition, the above-mentioned traditional network traffic situation prediction methods still have the following shortcomings: network traffic is a typical time series and has the characteristics of non-linearity, non-stationarity and time-dependence. These prediction methods are limited by the internal structure of the model and cannot well capture the long-term dependence in the time series.

At present, there are few traffic situation prediction methods in SDN (Hua et al., 2018). In Liu et al. (2021), a traffic situation prediction method based on RBFNN was proposed, which uses the SDN controller to collect network flow statistics, and uses RBFNN to analyze the nonlinear relationship of the network flow statistics to realize the traffic situation prediction. In Wang et al. (2021), a traffic situation prediction method based on ARMA was proposed. This method obtains network traffic information through the forwarding rules of the data, and combines ARMA to realize the traffic situation prediction. However, the above-mentioned method has the shortcomings of not fully considering the nonlinearity, non-stationarity, and long-term dependence of the network traffic. Therefore, the existing traffic situation prediction methods are no longer suitable for multi-domain SDN.

The gated recurrent unit (GRU) network is a typical deep learning model. Since the introduction of GRU, it has been widely used in the fields such as speech (Yuan, Tian & Lu, 2018), image (Mou, Ghamisi & Zhu, 2017), and natural language processing (Morchid, 2018). It has a great performance in solving complex and non-linear forecasting problems (Huang, Li & Huang, 2020), such as urban traffic flow prediction (Sun, Boukerche & Tao, 2020), energy consumption prediction (Kim & Cho, 2019), and urban rainfall prediction (Haidar & Verma, 2018). We propose GRU for traffic situation prediction in multi-domain SDN. The multi-domain SDN is dynamic and depends on many factors. If all factors are used for situation prediction, irrelevant and redundant information will greatly increase the computational complexity. In addition, the hyperparameters of the traditional GRU network are manually set, so that it consumes significant time and becomes difficult to select the most suitable hyperparameters. Based on these, we analyze the relevant factors affecting data traffic and control traffic; and use the salp swarm algorithm (SSA) to automatically optimize the hyperparameters of the GRU. Therefore, our contributions in this work can be summarized as follows:

1. We analyze the relevant factors that affect data traffic and control traffic, and transform them into a time series of actual situation values.
2. Manually setting hyperparameters of CNN and GRU has the disadvantage of consuming time and it is difficult to select the most suitable hyperparameters. The SSA is used to automatically optimize the hyperparameters of the GRU.
3. The traffic situation values in multi-domain SDN have more significant characteristics such as non-linearity, non-stationarity, and long-term dependence. Hyperparameters optimized GRU is used to predict the traffic situation values.

The rest of the article is organized as follows. The “Traffic situation factors” section analyzes the traffic situation factor. The “GRU hyperparameter optimization” section introduces the GRU hyperparameter optimization method. The “Multi-domain SDN traffic situation prediction” section explains multi-domain SDN traffic situation prediction method. The “Experiment and analysis” section simulates and analyzes the experimental results. Finally, the conclusions are given in the “Conclusions”.

## Traffic Situation Factors

Traffic is an important parameter to measure network operating load and status. To accurately predict the network status in the complex and changeable multi-domain SDN, various factors affecting traffic in it must fully be considered. In multi-domain SDN, there are two types of network traffic, including data traffic and control traffic (Shu et al., 2016). We analyze the factors that affect data traffic and control traffic, as well as extract them as situation factors. When the network equipment fails, broadcast packets are sent, which paralyzes network communication. Therefore, the values of the traffic situation factors, such as the average number of switch bytes and the number of lost packets, are calculated under the assumption that the network equipment does not fail.

### Data traffic factors

Data traffic refers to the traffic transmitted between switches. It exists in the switches and their communication links. Switch and link performance play a vital role in the transmission of data traffic (Tsai et al., 2018). Therefore, we extract factors that affect switch performance such as the average number of switch bytes and the ratio of the number of switch bytes to the number of switch packets (Sood, Yu & Xiang, 2016), as well as extract factors that affect link performance such as the number of the lost packets, the maximum transmission delay, and the average bandwidth utilization (Hu et al., 2020), as data traffic factors.

(1) The average number of switch bytes (ANB): the average number of bytes of all switches per unit time. It is defined as follows:

(1) $$ANB = \displaystyle{1 \over {TN}}\sum\limits_{i = 1}^N {bytes\_nu{m_i}}$$

In Eq. (1), $T$ is the sampling period, $bytes\_nu{m_i}$ is the number of bytes of the ${i^{th}}$ switch, and $N$ is the number of switches.

(2) The ratio of the number of switch bytes to the number of switch packets (RBP): the ratio of the number of bytes to the number of packets of all switches. It is defined as follows:

(2) $$RBP = \displaystyle{{\sum\limits_{i = 1}^N {bytes\_nu{m_i}} } \over {\sum\limits_{i = 1}^N {packets\_nu{m_i}} }}$$

where $packets\_nu{m_i}$ is the number of packets of the ${i^{th}}$ switch.

(3) The number of lost packets (NLP): the number of lost and errored packets in the network per unit time. It is calculated as follows:

(3) $$NLP = \displaystyle{{packet\_nu{m_{loss}}} \over T}$$

where $packet\_nu{m_{loss}}$ is the number of lost and errored packets, communicating between switches. The number of lost packets can characterize the network's service quality. If the number of lost and errored packets is smaller, the service quality of the network will be better. When 1,000 packets are sent, the number of lost packets is less than 10, preferably 0.

(4) The maximum transmission delay (MTD): the maximum transmission delay between the sending port and the receiving port of all links in the sampling period. It is calculated as follows:

(4) $$MTD = \max \left\{ {portdelay\left( i \right)} \right\}$$

where $portdelay\left( i \right)$ represents the transmission delay from the sending port to the receiving port of the ${i^{th}}$ link. The maximum transmission delay can better characterize the transmission quality of the network. If the delay is very large at that time, it will mean that congestion has occurred on some links.

(5) The average bandwidth utilization (ABU): The average value of the link load on all links to the link bandwidth. It is calculated as follows:

(5) $$ABU = \displaystyle{1 \over L}\sum\limits_{i = 1}^L {\displaystyle{{bytes\_transmi{t_i}} \over {bandwidt{h_i}}}}$$

where $bandwidt{h_i}$ represents the bandwidth of the ${i^{th}}$ link, $bytes\_transmi{t_i}$ represents the number of bytes transmitted per unit time on the ${i^{th}}$ link, and $L$ represents the total number of links.

### Control traffic factors

Control traffic refers to the traffic transmitted between the controllers and the switches. Mainly through *Packet_in* and *Flow_mod* packets, the controllers communicate with the switches. Therefore, we extract the average numbers of *Packet_in* and *Flow_mod* packets as control traffic factors.

(1) The average number of *Packet_in* (ANP): The average number of *Packet_in* packets interacted by all switches and controllers per unit time. It is calculated as follows:

(6) $$ANP = \displaystyle{1 \over {TN}}\sum\limits_{i = 1}^N {Packet\_in\_num\left( i \right)}$$

where $Packet\_in\_num\left( i \right)$ represents the number of *Packet_in* packets transmitted by the ${i^{th}}$ switch. When a large number of *Packet_in* packets appear, excessive occupation of resources of controller and switch causes network failures.

(2) The average number of *Flow_mod* (ANF): the average number of *Flow_mod* packets interacted by all switches and controllers per unit time. It is calculated as follows:

(7) $$ANF = \displaystyle{1 \over {TN}}\sum\limits_{i = 1}^N {Flow\_ {\rm mod} \_num\left( i \right)}$$

where $Flow\_{\rm mod} \_num\left( i \right)$ represents the number of *Flow_mod* packets transmitted by the ${i^{th}}$ switch. Similar to the above equation, when a large number of *Flow_mod* packets appear, excessive occupation of controller and switch resources causes network failures.

This article refers to the quantitative calculation method of literature (Hong, Fessler & Balzano, 2018). The specific process is as follows: first, carry out the min-max normalization coding for the above situation factor values (Cerda & Varoquaux, 2020); then, according to the importance of each factor, assign different weights to the factors; finally, obtain the traffic situation values through weighted calculation of the normalized values of situation factors. The traffic situation values (TSV) are calculated as follows:

(8) $$\eqalign{& TSV = {\omega _{ANB}} * AN{B_{normal}} + {\omega _{RBP}} * RB{P_{normal}} + {\omega _{NLP}} * NL{P_{normal}} \cr & + {\omega _{MTD}} * MT{D_{normal}} + {\omega _{ABU}} * AB{U_{normal}} + {\omega _{ANP}} * AN{P_{normal}} + {\omega _{ANF}} * AN{F_{normal}}}$$

where $AN{B_{normal}}$, $RB{P_{normal}}$, $NL{P_{normal}}$, $MT{D_{normal}}$, $AB{U_{normal}}$, $AN{P_{normal}}$ and $AN{F_{normal}}$ are the normalized values of situation factors; ${\omega _{ANB}}$, ${\omega _{RBP}}$, ${\omega _{NLP}}$, ${\omega _{MTD}}$, ${\omega _{ABU}}$, ${\omega _{ANP}}$ and ${\omega _{ANF}}$ are the weights of the corresponding situation factors. Among them, the principal component analysis (PCA) is adopted to determine the weights (Tao et al., 2019). This method can learn the laws from historical data more scientifically and objectively, and reduce the interference of people’s subjective opinions (Jolliffe & Cadima, 2016). Therefore, the obtained traffic situation values can more accurately represent the current network state.

Under different network operating states, the network traffic situation values correspond to different division results. Generally, it can be divided into four levels of network status according to expert’s experience (Bhatia et al., 2020), as shown in Table 1.

**Table 1:** Multi-domain SDN network situation level.

Traffic situation value	Description
0~0.25	The network is in a state of low utilization, with less business flow
0.25~0.50	The network is in a high utilization state and it is in good condition
0.50~0.75	The network is busy and maybe congested
0.75~1.0	The network is at a higher risk and may face paralysis

## GRU Hyperparameter Optimization

The gated recurrent unit (GRU) network is good at processing time series. It uses multiple processing layers to approximate arbitrary complex functions and captures long-term dependence of time series by using multiple gates. Multi-domain SDN network traffic situation values have the characteristics such as non-linearity, long-term dependence, and non-stationarity, which is very suitable for using the GRU network to predict traffic situation values. The hyperparameters have a greater impact on the prediction accuracy of the GRU network. The hyperparameters of the traditional GRU network are manually optimized, which has the disadvantage of time-consuming and being difficult to select the most suitable hyperparameters. To improve the prediction effect, we use the SSA to automatically optimize the GRU hyperparameters.

### Key GRU hyperparameters

**Figure 1:**
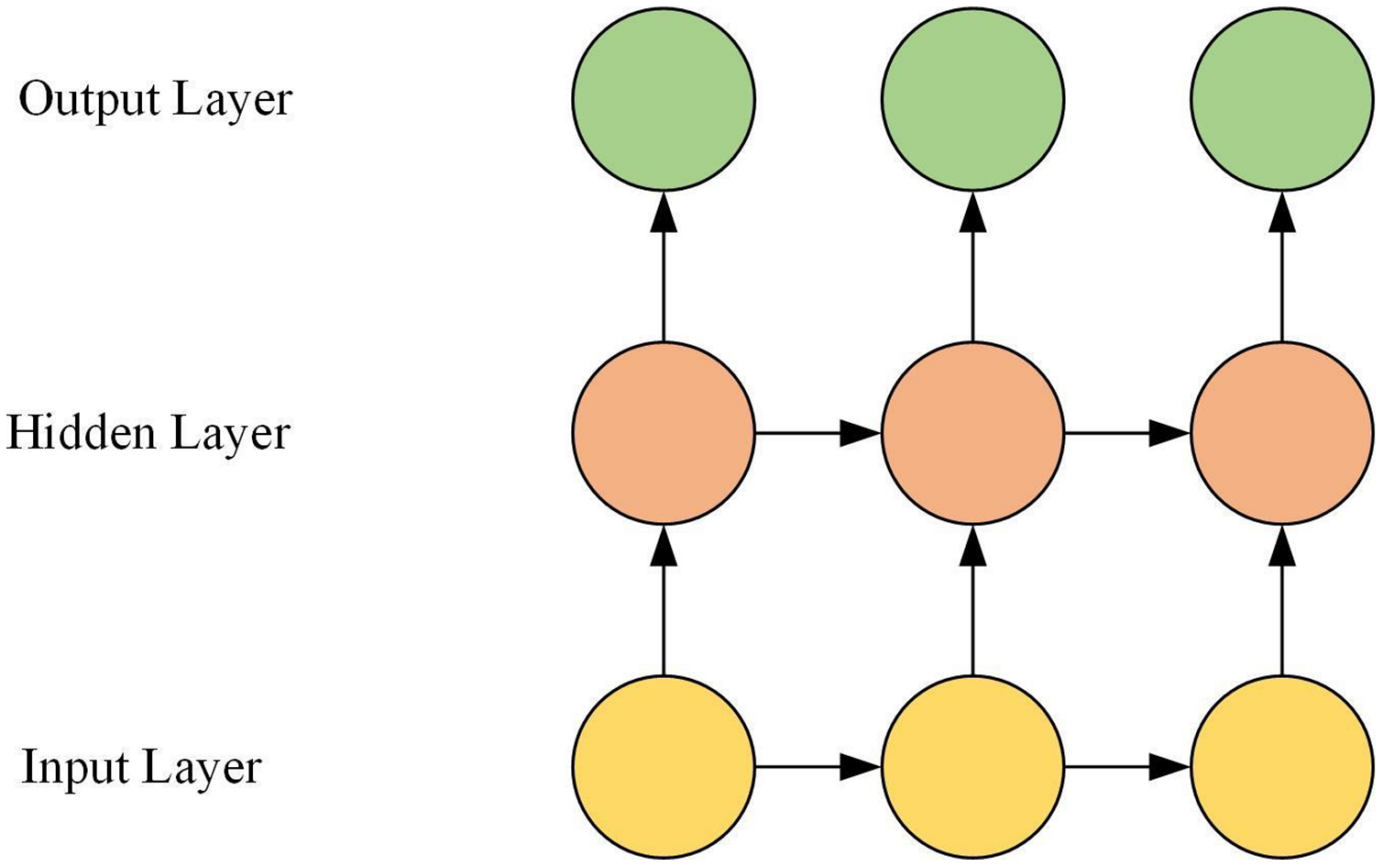
GRU network structure.

The GRU is composed of the input layer, the hidden layer, and the output layer. The hidden layer is composed of multiple GRU neurons. The GRU network structure is shown in Fig. 1.

The prediction effect of GRU mainly depends on the hyperparameters. The key hyperparameters of GRU are shown in Table 2.

1. The number of hidden layers and neurons in each hidden layer are important hyperparameters of GRU. If the number of hidden layers and GRU neurons in each hidden layer are larger, the network’s expression ability and fitting ability will be stronger, and the prediction effect will be better. On the contrary, overfitting will be likely to occur.
2. The window size is to predict the value of the next moment based on how much historical data is present. If the window size is too small (large), it will not reflect true traffic situation, and the prediction effect will be poor.

**Table 2:** Key GRU hyperparameters.

Hyperparameters	Type	Selection interval
The number of hidden layers	Discrete	Integer range [1,5]
The number of GRU neurons in each hidden layer	Discrete	Integer range [20,70]
Window size	Discrete	Integer range [2,10]

### GRU hyperparameters optimized by SSA

The Salp Swarm Algorithm (SSA) is a heuristic algorithm that simulates the foraging of salps in the biological world (Mirjalili et al., 2017). Compared with other heuristic algorithms such as the Particle Swarm Optimization (PSO) and the Firefly Algorithm (FA), it has various advantages such as high optimization accuracy, good robustness, and fast convergence speed. SSA divides the population into leaders and followers. The leader leads the followers, which forms the salp chain to perform population optimization. As the leader leads the followers, the entire population quickly and accurately converges to the optimal solution of the problem. To improve the prediction accuracy, we adopt SSA to automatically optimize the hyperparameters of the GRU network. The specific idea is as follows: the position of each salp individual represents a kind of hyperparameter configuration of the GRU network. Through the iterative optimization of salp individuals, the individual position with the best fitness value is the optimal hyperparameter configuration of the GRU network.

**Figure 2:**
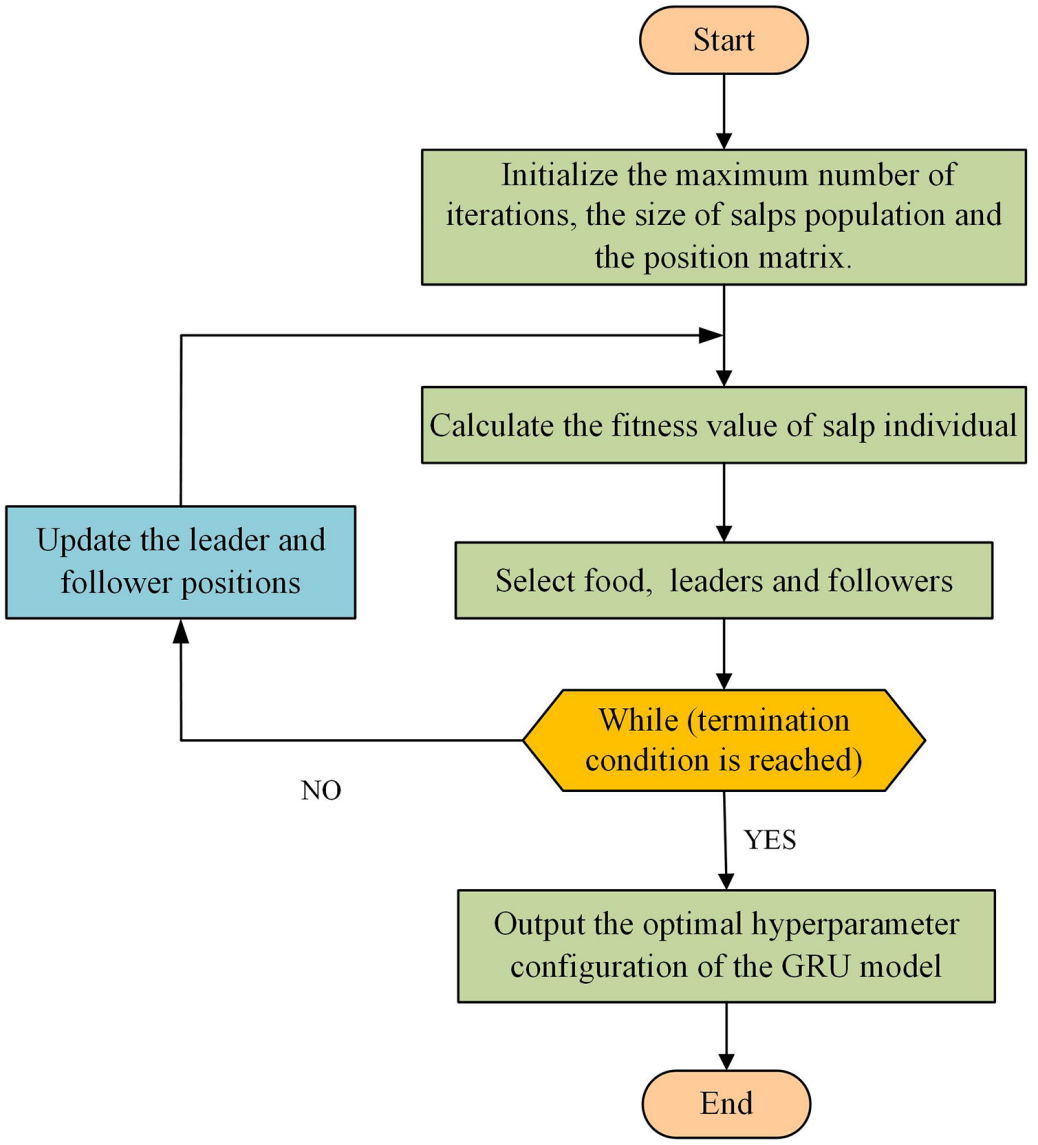
Flowchart of GRU hyperparameters optimized by SSA.

The specific steps are as follows, and the flowchart is shown in Fig. 2.

**Step 1** Initialize the maximum number of iterations $L$, the size of the salp population $N$, and the position matrix ${X_{N \times D}}$.

**Step 2** Calculate the fitness value of salp individuals in the population.

**Step 3** Select the position of the food. The salp individuals in the population are sorted according to their fitness values, and the salp position with the best fitness value is regarded as the food position.

**Step 4** Select the position of the leaders and followers. After selecting the food position, there are $N - 1$ salp individuals remaining in the population. The salps with the top half of the fitness value are regarded as the leaders and the rest as the followers.

**Step 5** Update the leader and follower positions according to the literature (Mirjalili et al., 2017).

**Step 6** Determine whether the maximum number of iterations $L$ is reached. If it is reached, the individual position with the best fitness value is taken as the optimal hyperparameter configuration of the GRU model. Otherwise, go to **Step 2**.

## Multi-Domain SDN Traffic Situation Prediction

**Figure 3:**
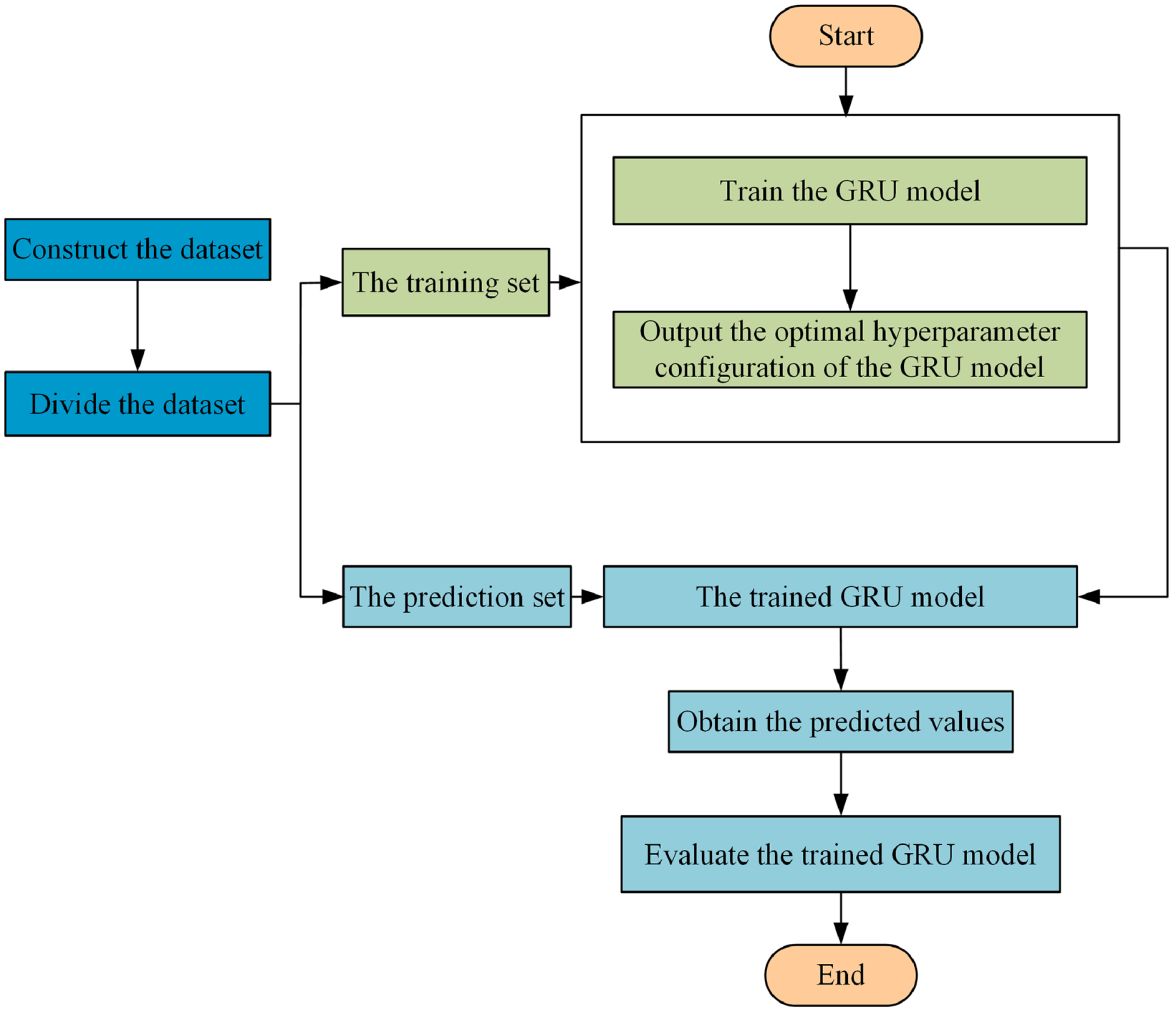
Flowchart of multi-domain SDN traffic situation prediction.

In this paper, GRU optimized by SSA is applied to the traffic situation prediction in multi-domain SDN. The historical traffic situation values are used as the input of the model, to predict the traffic situation value at the next moment. The specific steps of the traffic situation prediction in multi-domain SDN are as follows, and the specific flowchart is shown in Fig. 3.

**Step 1** Construct the dataset. According to the window size $\tau$, the time series $X = \left( {{x_1},{\kern 1pt} {\kern 1pt} {\kern 1pt} {x_2},{\kern 1pt} {\kern 1pt} {\kern 1pt} \cdot \cdot \cdot {\kern 1pt} {\kern 1pt} {\kern 1pt} ,{\kern 1pt} {\kern 1pt} {\kern 1pt} {x_n}} \right)$ of actual multi-domain SDN situation values is used to construct the dataset. The number of samples in the dataset is $n - \tau$. The construction results of the dataset are shown in Table 3.

**Table 3:** Construction results of the dataset.

Sample number	Input data	Output data
1	${x_1,\;x_2,\;\cdots,\;x_\tau}$	${x_{\tau+1}}$
2	${x_2,\;x_3,\;\cdots,\;x_{\tau+1}}$	${x_{\tau+2}}$
$\cdots$	$\cdots$	$\cdots$
${n-\tau}$	${x_{n-\tau},\;x_{n-\tau+1},\;\cdots\;x_{n-1}}$	${x_n}$

**Step 2** Divide the dataset. The dataset in **Step 1** is divided into the training set and prediction set. The training set is used to train the GRU model, and the prediction set is used to verify the effectiveness of the trained model.

**Figure 4:**
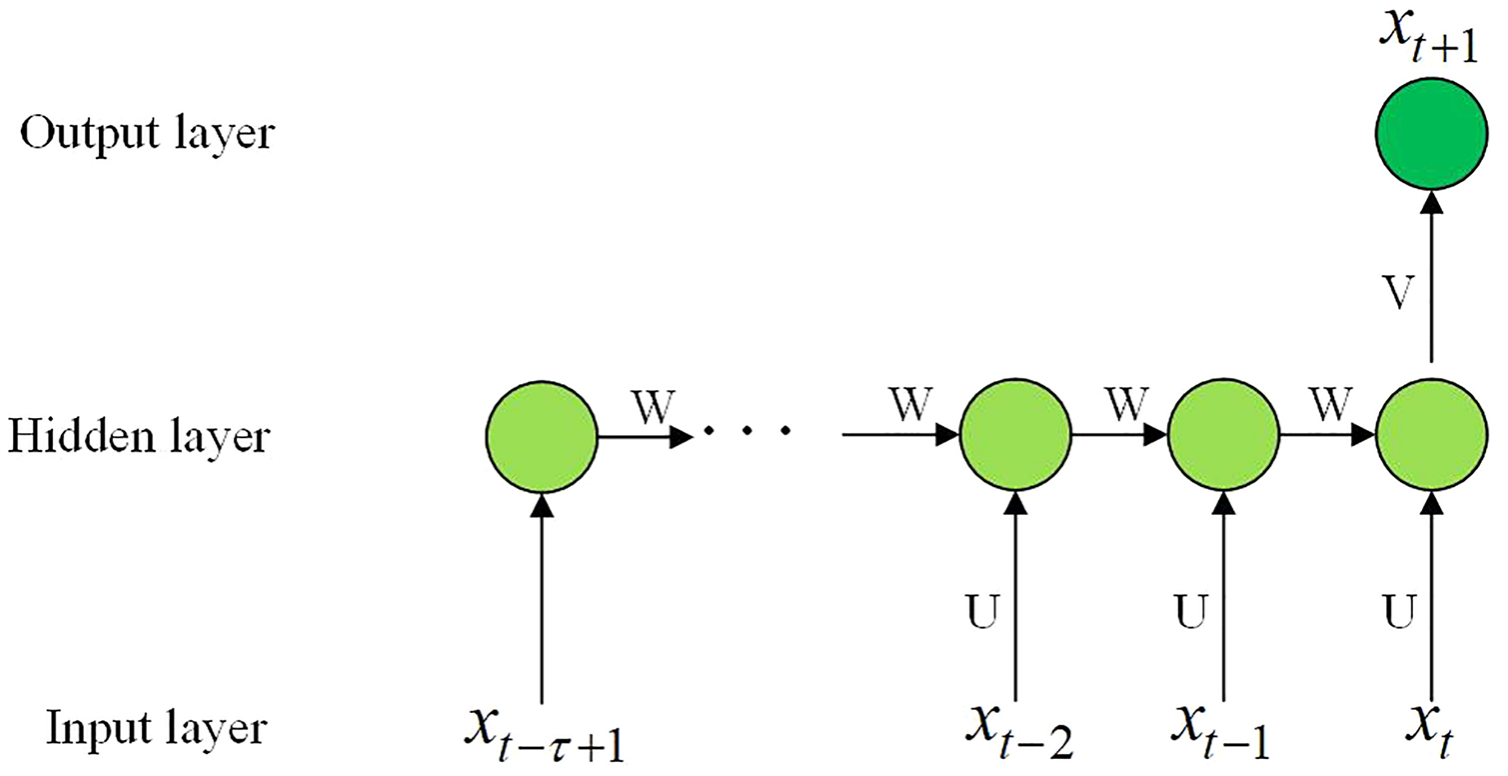
The GRU prediction model.

**Step 3** Train the GRU model. The GRU prediction model is shown in Fig. 4.

1. Initialize the GRU hyperparameters: the number of hidden layers, the number of GRU neurons in each hidden layer, and the step size.
2. Initialize the salp population: the maximum number of iterations $L$, the size of the salp population $N$, and the position matrix ${X_{N \times D}}$.
3. According to the training set and fitness function, the fitness value of the salp individual is calculated. The positions of salp individuals are updated iteratively, and the salp individual position with the best fitness value is the optimal hyperparameter configuration of the GRU model. The root mean square error of the predicted values and the actual values is taken as the fitness function. It is calculated as follows (Buche, Schraudolph & Koumoutsakos, 2005):

(9) $$fitness\left( i \right) = \sqrt {\displaystyle{1 \over m}\sum\limits_{i = 1}^m {{{\left( {{y}^{\prime} - y} \right)}^2}} }$$

where $m$ is the number of samples in the training set, ${y}^{\prime}$ is the output value of GRU, and $y$ is the real value. When the fitness value of the individual salp position is the smallest, it is taken as the GRU model with the optimal hyperparameter.

**Step 4** The predicted values are obtained by inputting the data in the prediction set into the trained GRU network. The predicted value is compared with the actual value, and the prediction accuracy of the model is measured by the evaluation index.

## Experiment and Analysis

**Figure 5:**
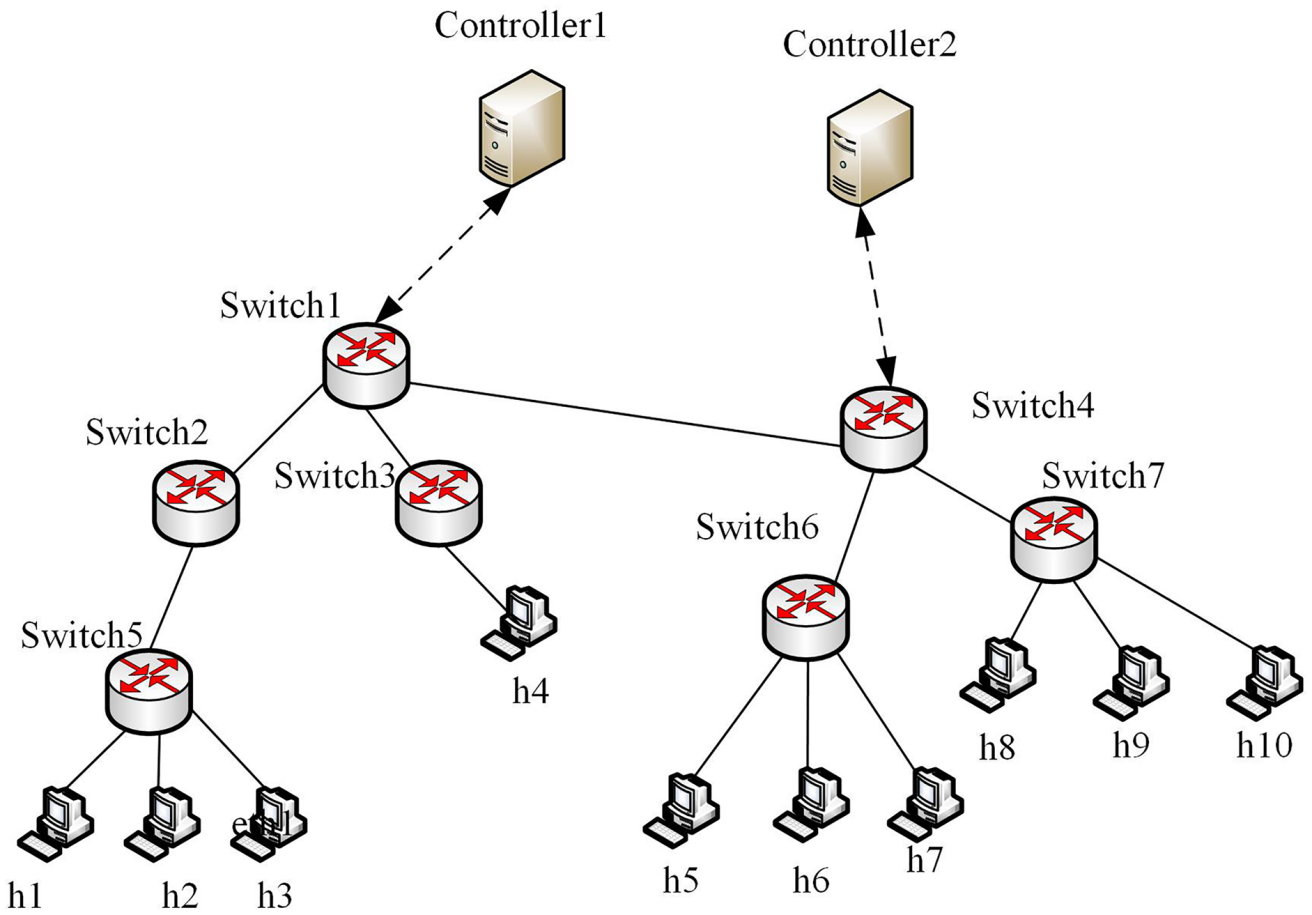
Multi-domain SDN network topology.

We used Mininet to construct the SDN network, where the controller uses the open-source Python-based controller Ryu. The deep learning module is developed based on the Keras framework, and the operating system environment is Ubuntu 16.04. A built multi-domain SDN platform, that can effectively simulate the real network environment, is composed of the SDN_1 domain and SDN_2 domain. There are two controllers, seven switches, and 10 hosts. The experimental topology is shown in Fig. 5.

**Figure 6:**
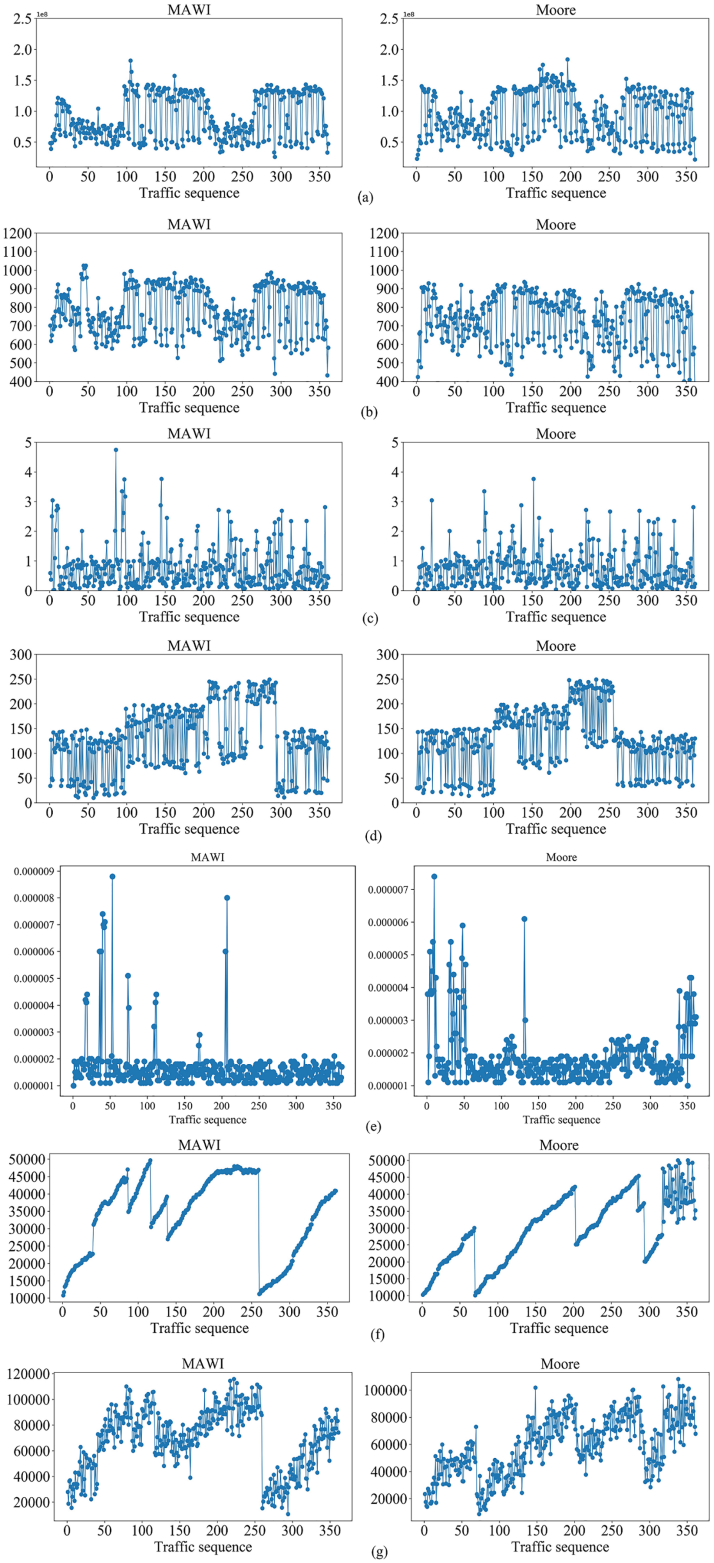
Changes of the situation factor values over time on MAWI and Moore datasets, respectively. (A) The average number of switch bytes (bps). (B) The ratio of the number of switch bytes to the number of switch packets. (C) The number of lost packets (s). (D) The maximum transmission delay (pps). (E) The average bandwidth utilization. (F) The average number of Packet_in (pps). (G) The average number of Flow_mod (pps).

To demonstrate the effectiveness of the proposed method, we selected public traffic datasets MAWI2019 (Blaise et al., 2020) and Moore2003 (Moore & Zuev, 2005) as our experiment datasets. The two datasets contain raw traffic data, and the publication years, data terminals and IP addresses differ greatly. Thus, they can be used to effectively evaluate the generality of the proposed method. The traffic in MAWI and Moore datasets were injected into multi-domain SDN in Fig. 5, respectively, to simulate the traffic in the datasets. By extracting the situation factors in the “Traffic situation factors” section, the corresponding values are obtained. For the MAWI and Moore datasets, the changes of the situation factor values of multi-domain SDN over time are shown in Fig. 6.

**Figure 7:**
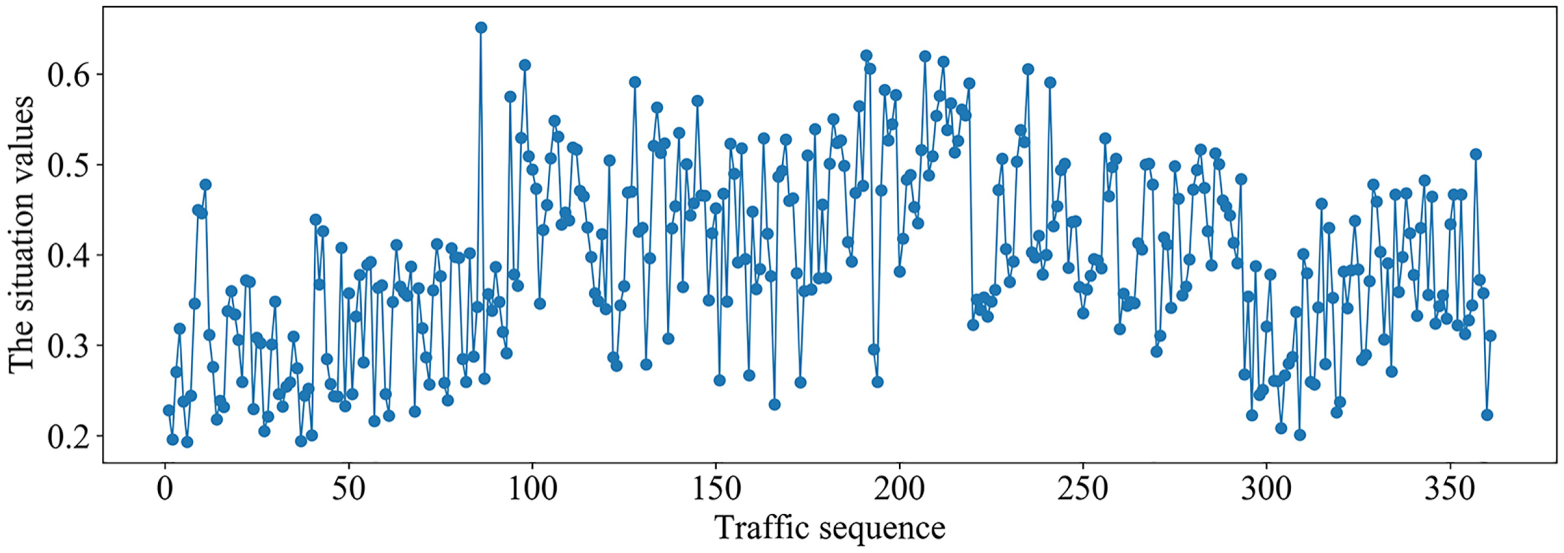
Traffic situation values on MAWI dataset.

**Figure 8:**
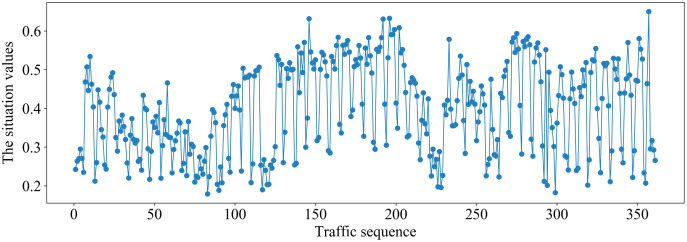
Traffic situation values on the Moore dataset.

It can be seen from Fig. 6 that the different situation factors related to traffic in the multi-domain SDN have quite different value ranges and changing trends. According to the literature (Hong, Fessler & Balzano, 2018), the traffic situation values on MAWI and Moore datasets are calculated. The traffic situation values on MAWI and Moore datasets are shown in Figs. 7 and 8.

It can be seen from Fig. 7 that traffic situation values are relatively high and there is more business traffic when the traffic sequence is between 100–290 on MAWI dataset. The network situation is in a stable state when the traffic sequence is between 0–100 or 290–350.

It can be seen from Fig. 8 that the traffic situation values are relatively high and there is more business traffic when the traffic sequence is between 100–200 or 270–350 on Moore dataset. The network situation is in a stable state when the traffic sequence is between 0–100 or 200–270.

To verify the effectiveness of the proposed method, we used the traffic situation values on MAWI and Moore to construct the datasets according to Step 1 of section “Multi-domain SDN traffic situation prediction”, respectively. The dataset constructed from the traffic situation values on MAWI is named as dataset_1. The dataset constructed from the traffic situation values on the Moore is named as dataset_2. To enable the model to be better trained and improve the model prediction accuracy, both dataset_1 and dataset_2 are divided into a training set and prediction set according to a ratio of 8:2. The training set is adopted to train the model, and the prediction set is used to detect the prediction effect of the trained model.

**Figure 9:**
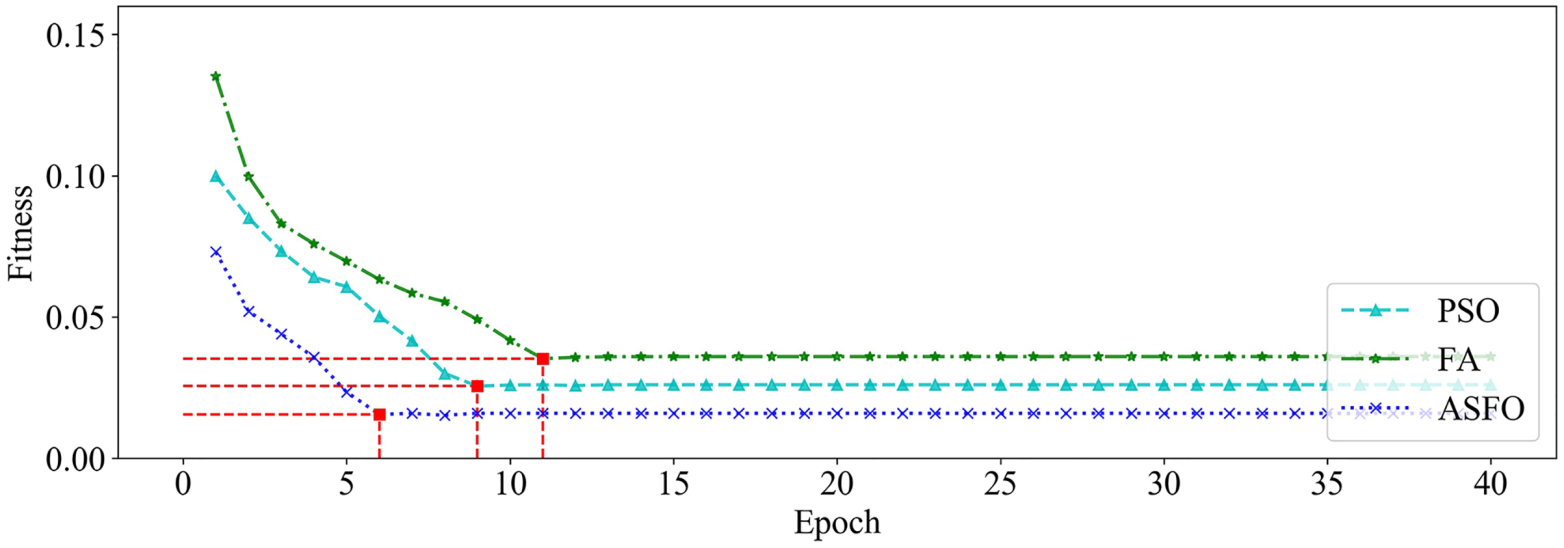
The changing curve of fitness value on dataset_1.

**Figure 10:**
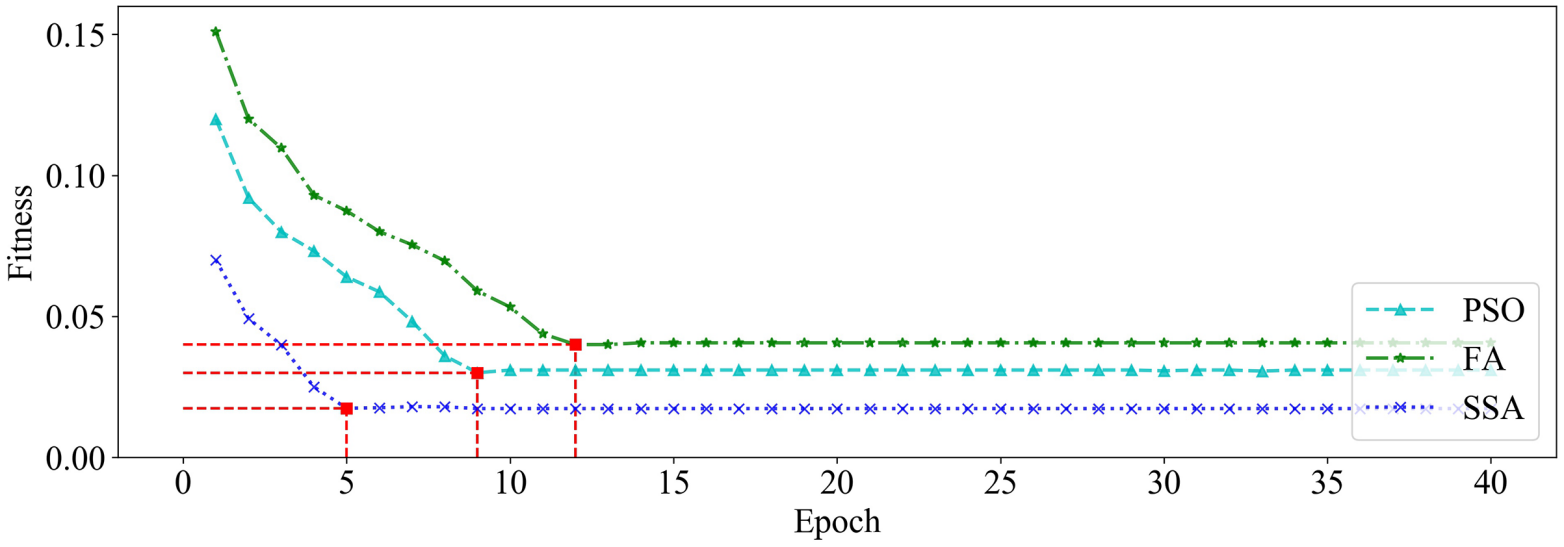
The changing curve of fitness value on dataset_2.

We utilized PSO, FA and SSA, respectively, to optimize the GRU hyperparameters on dataset_1 and dataset_2. Among them, the size of the population is set to 20, and the maximum number of iterations is set to 40. The changing curves of fitness values of the three algorithms in the iterative process are shown in Figs. 9 and 10, respectively.

It can be seen from Figs. 9 and 10 that, compared with PSO and FA, the SSA has better fitness values and converges faster during the iteration process.

**Figure 11:**
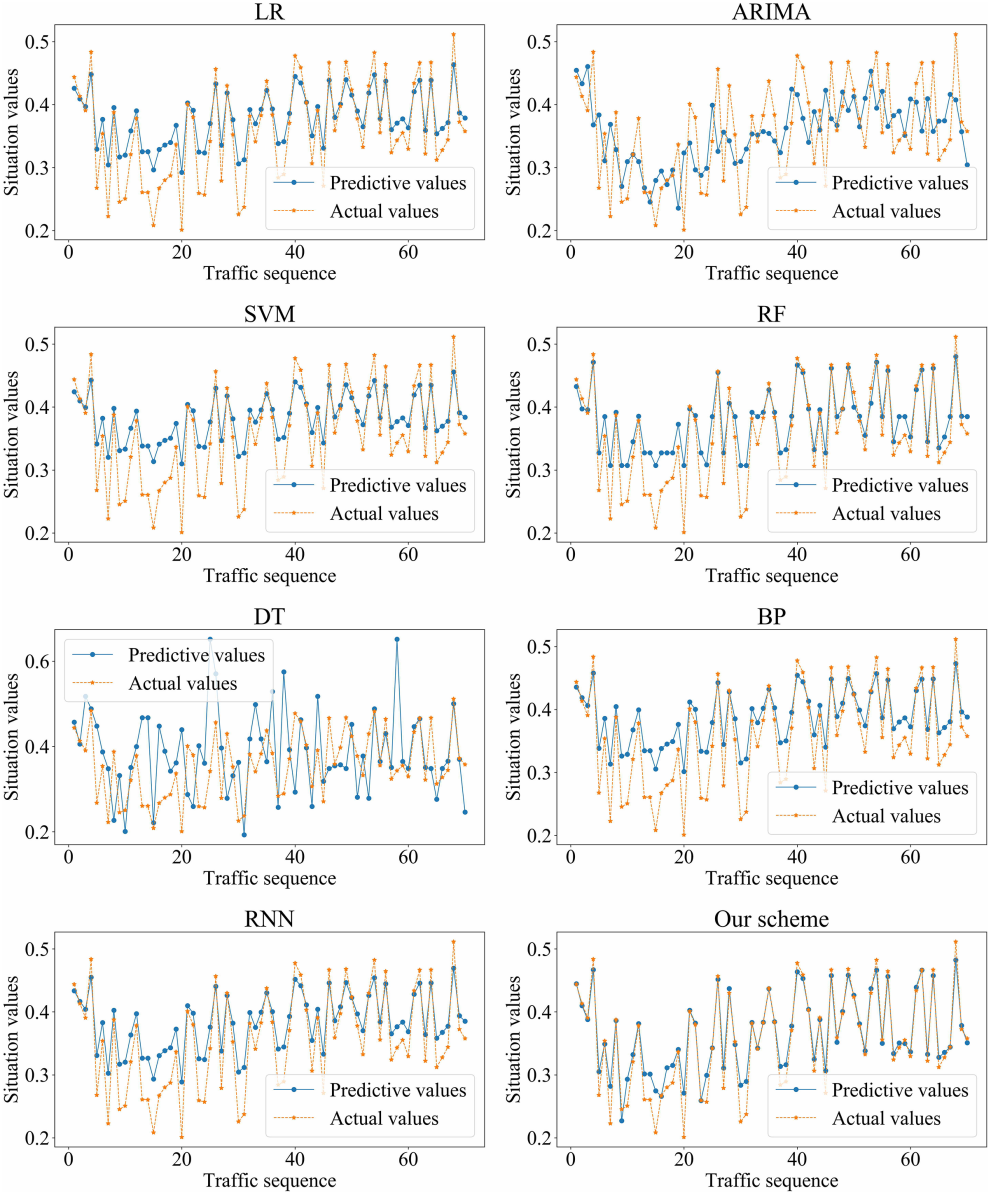
Prediction results of different algorithms on dataset_1.

**Figure 12:**
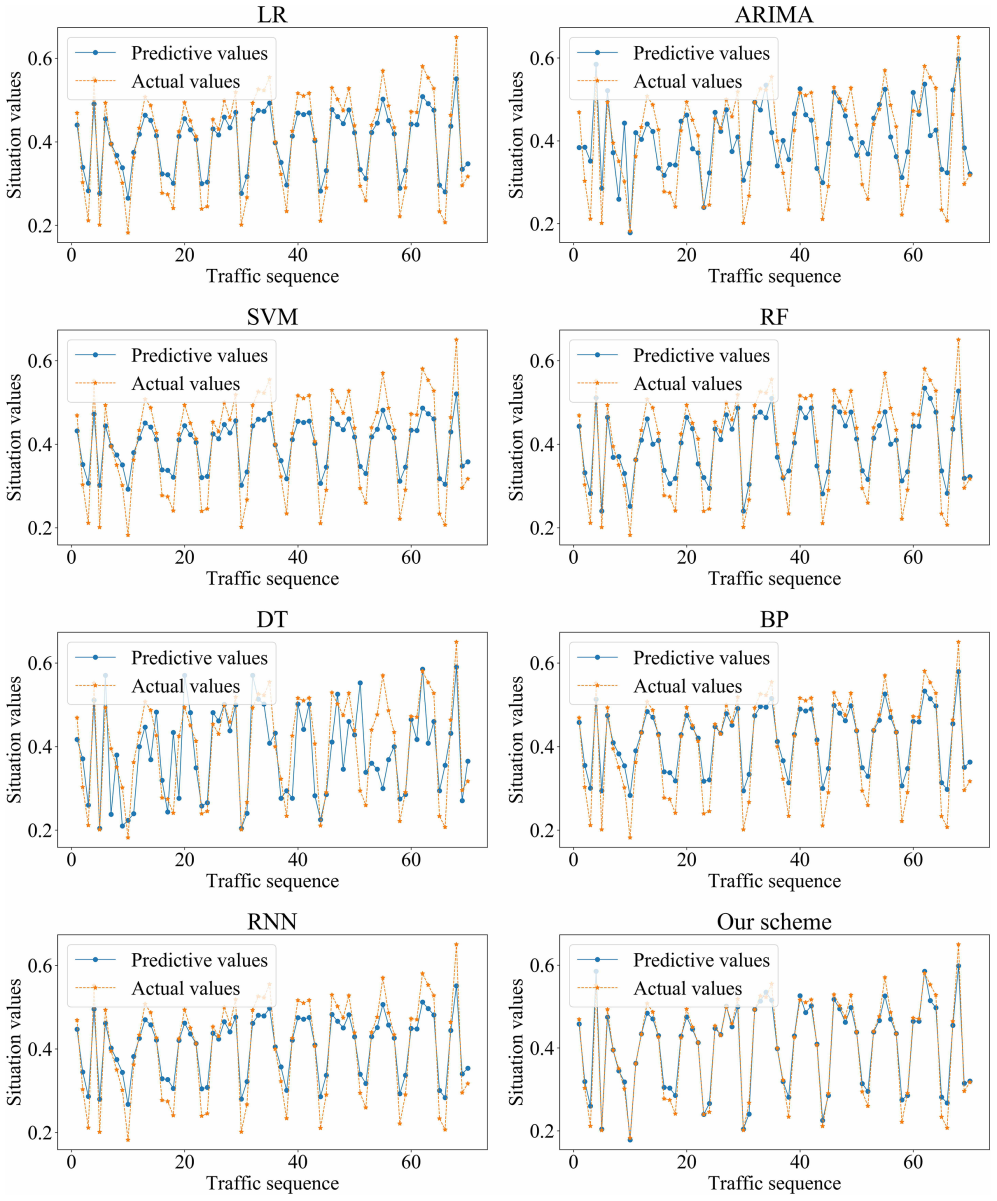
Prediction results of different algorithms on dataset_2.

We adopted LR, ARIMA, SVM, RF, DT, BP, RNN, and our scheme (*i.e.*, SSA-GRU), respectively, to forecast the traffic situation values on dataset_1 and dataset_2. The optimal GRU hyperparameters selected by SSA on dataset_1 and dataset_2 are shown in Tables 4 and 5, respectively. The prediction results of each algorithm on dataset_1 and dataset_2 are shown in Figs. 11 and 12, respectively.

**Table 4:** Optimal GRU hyperparameters on the dataset_1.

Hyperparameter	Selection
Number of hidden layers	3
Number of GRU neurons in the first hidden layer	38
Number of GRU neurons in the second hidden layer	35
Number of GRU neurons in the third hidden layer	27
Window size	6

**Table 5:** Optimal GRU hyperparameters on the dataset_2.

Hyperparameter	Selection
Number of hidden layers	3
Number of GRU neurons in the first hidden layer	40
Number of GRU neurons in the second hidden layer	33
Number of GRU neurons in the third hidden layer	28
Window size	6

In addition, the root mean square error (RMSE), average absolute error (MAE), average relative error (MRE) (Wang et al., 2018) and mean absolute percentage error (MAPE) were used to evaluate the prediction performance of different algorithms. The formulas are defined as follows:

(10) $$RMSE = \sqrt {\displaystyle{1 \over M}\sum\limits_{i = 1}^M {{{\left( {{{{y}^{\prime}}_i} - {y_i}} \right)}^2}} }$$

(11) $$MAE = \displaystyle{1 \over M}\sum\limits_{i = 1}^M {\left| {{{{y}^{\prime}}_i} - {y_i}} \right|}$$

(12) $$MRE = \displaystyle{1 \over M}\sum\limits_{i = 1}^M {\displaystyle{{\left| {{{{y}^{\prime}}_i} - {y_i}} \right|} \over {{y_i}}}}$$

(13) $$MAPE = \displaystyle{1 \over M}\sum\limits_{i = 1}^M {\displaystyle{{\left| {{{{y}^{\prime}}_i} - {y_i}} \right|} \over {{y_i}}} \times 100\% }$$

where $M$ is the number of samples in the dataset, ${y}^{\prime}$ is the output value of GRU, and $y$ is the real value.

The prediction performance of different algorithms on dataset_1 and dataset_2 is shown in Tables 6 and 7, respectively.

**Table 6:** Prediction performance of different algorithms on dataset_1.

Algorithms	RMSE	MAE	MRE	MAPE/%
LR	0.0413	0.0341	0.1151	11.51%
ARIMA	0.0786	0.0665	0.2010	20.10%
SVM	0.0493	0.0408	0.1380	13.80%
RF	0.03885	0.0299	0.1048	10.48%
DT	0.1244	0.0992	0.3091	30.91%
BP	0.04676	0.0386	0.1322	13.22%
RNN	0.0422	0.0354	0.12	12%
SSA-GRU	0.0234	0.0155	0.0563	5.63%

**Table 7:** Prediction performance of different algorithms on dataset_2.

Algorithms	RMSE	MAE	MRE	MAPE/%
LR	0.0465	0.0413	0.1275	12.75%
ARIMA	0.0736	0.0624	0.1863	18.63%
SVM	0.0613	0.0542	0.1627	16.27%
RF	0.0498	0.0430	0.1271	12.71%
DT	0.087	0.0653	0.1742	17.42%
BP	0.0471	0.0372	0.1275	12.75%
RNN	0.0461	0.0397	0.1229	12.29%
SSA-GRU	0.0226	0.0162	0.0487	4.87%

Based on the above experiment results, the proposed method has better prediction accuracy and prediction effect. The prediction error is smaller. The predictive and actual values fit better.

## Conclusions

To predict the multi-domain SDN traffic status comprehensively and accurately, we proposed a traffic situation prediction method based on the GRU network in multi-domain SDN. First, we analyzed the relevant factors that affect data traffic and control traffic, and convert them into a time series of actual situation values. Then, to overcome the shortcomings of manually optimizing the hyperparameters of GRU such as time-consuming and difficulty in selecting the most suitable hyperparameters, we used SSA to automatically optimize the hyperparameters of GRU, which improves the prediction effect. Finally, we used hyperparameter optimized GRU to predict the situation values. The results of multiple experiments on the traffic datasets MAWI and Moore show that the proposed method has higher prediction accuracy than traditional machine learning algorithms. Limited by the experimental conditions, only two domains without network failures have been tested. In future work, further research will be conducted on three and more domains with network failures. To better verify the prediction effect of our proposed method, we will deploy the proposed method in a real environment and explore more application scenarios.

## Supplemental Information

10.7717/peerj-cs.1011/supp-1 Supplemental Information 1 Situation code.

## Additional Information and Declarations

### Data Availability

The following information was supplied regarding data availability: The Moore dataset is available at figshare: kuan, james (2022): Moore dataset. figshare. Dataset. https://doi.org/10.6084/m9.figshare.18467507.v1. The NISM dataset is available at figshare: kuan, james (2022): NISM dataset.zip. figshare. Dataset. https://doi.org/10.6084/m9.figshare.18467504.v1.
